# Trends in and factors associated with late initiation of antiretroviral therapy among newly diagnosed HIV cases, Kampong Thom, Cambodia, 2014–2023

**DOI:** 10.5365/wpsar.2025.16.4.1207

**Published:** 2025-12-24

**Authors:** Vathanak Sann, Sengdoeurn Yi, Chanratana Leng, Sophanith Ung, Khemrin Pong

## Abstract

Late initiation of antiretroviral therapy (ART) is associated with worse health outcomes for people living with HIV. In 2019, Cambodia implemented a same-day policy allowing people with HIV to start ART on the day they were diagnosed. Using case data collected by Cambodia’s National Center for HIV/AIDS Dermatology and STDs, this study examined trends and factors influencing late ART initiation among newly diagnosed HIV cases attending three clinics in Kampong Thom province from 2014 to 2023. Factors linked to late ART initiation (defined as starting treatment > 1 day after diagnosis) were assessed using descriptive statistics and logistic regression. Statistical significance was set at *P* < 0.05. The study included 741 newly diagnosed HIV cases, with a mean age of 45.7 years at diagnosis (standard deviation: 10.2); 57.1% (423) of cases were female and more than 70% (504/704) were employed. In the 5-year period before the implementation of same-day ART initiation (2014–2018), 91% (325/356) of cases had late ART initiation. In the subsequent 5-year period (2019–2023), the proportion of cases with late ART initiation averaged 23%, and in 2023 was just 3%. Late ART initiation was more common at the Baray Santuk clinic (adjusted odds ratio: 3.94, 95% confidence interval: 1.99–7.81, *P* < 0.001), likely due to a lack of staff dedicated to HIV care. The findings demonstrate that same-day ART initiation is feasible in resource-limited settings and that adequate staffing can further improve prompt ART initiation.

HIV remains a significant public health concern worldwide. As of the end of 2023, an estimated 39.9 million individuals were living with HIV, and during 2023, 1.3 million became newly infected. (*1*) In the Asia-Pacific region alone, in 2023, there were an estimated 6.6 million people living with HIV and around 300 000 new infections. (*1*, *2*) The effectiveness of HIV prevention, diagnosis, treatment and care programmes varies considerably across regions, countries and populations.

The World Health Organization (WHO) currently recommends rapid initiation of antiretroviral therapy (ART) for all patients newly diagnosed with HIV, with same-day initiation for those ready to start treatment. (*3*) The United States Department of Health and Human Services also recommends immediate ART initiation. (*4*) This approach aims to increase ART uptake, reduce the time to viral suppression and improve virological suppression rates. (*5*)

As of December 2022, the latest available data showed that an estimated 76 000 people in Cambodia are living with HIV. Since 1991, a total of 65 587 cases of HIV have been reported, of whom 99% have accessed ART. Of those who received ART, 98% achieved viral suppression. (*6*) In 2019, Cambodia’s National Center for HIV/AIDS Dermatology and STDs advised all of its ART sites to implement a same-day policy by offering patients the option of starting ART on the day that their diagnosis of HIV was confirmed. The same-day policy was introduced with the dual aim of improving links to care and reducing the risk of loss to follow up, and enhancing treatment outcomes for people living with HIV. (*7*)

To be eligible to start same-day ART, patients must have a confirmed HIV diagnosis and have completed a clinical readiness assessment. This assessment takes the form of a clinical evaluation by a health-care provider to determine whether the patient is medically stable. It typically includes symptom screening for serious opportunistic infections, a basic physical examination, a review of medical history and a set of baseline laboratory tests, ideally CD4 count, creatinine and haemoglobin levels, and liver function tests. Provided the patient is deemed clinically stable and is free of opportunistic infections, such as tuberculosis or cryptococcal meningitis, ART can be initiated immediately without waiting for blood test results. The new policy was designed to offer a more streamlined approach to providing ART by reducing some of the procedural barriers to initiating treatment.

Since the implementation of the same-day policy in Cambodia, no study has measured its impact on ART initiation rates. This study aims to examine the trends in, and determinants of, late ART initiation among patients newly diagnosed with HIV in Kampong Thom, a province in central Cambodia, using HIV case data routinely collected by the National Center for HIV/AIDS Dermatology and STDs. Kampong Thom province was selected due to its combination of a moderate HIV burden and programmatic relevance. It provides a representative setting in which to assess policy implementation in a semi-rural context where challenges such as delayed diagnosis, stigma and service accessibility remain key barriers to early ART initiation. Findings from this setting may offer insights that are applicable to similar provinces across the country.

## Methods

### Study setting

Kampong Thom province has nine districts, and it borders the provinces of Siem Reap, Preah Vihear, Stung Treng, Kratie, Kampong Cham, Kampong Chhnang and Tonle Sap. It is Cambodia’s second largest province by area and has a population of around 750 000.

**Fig. 1:**
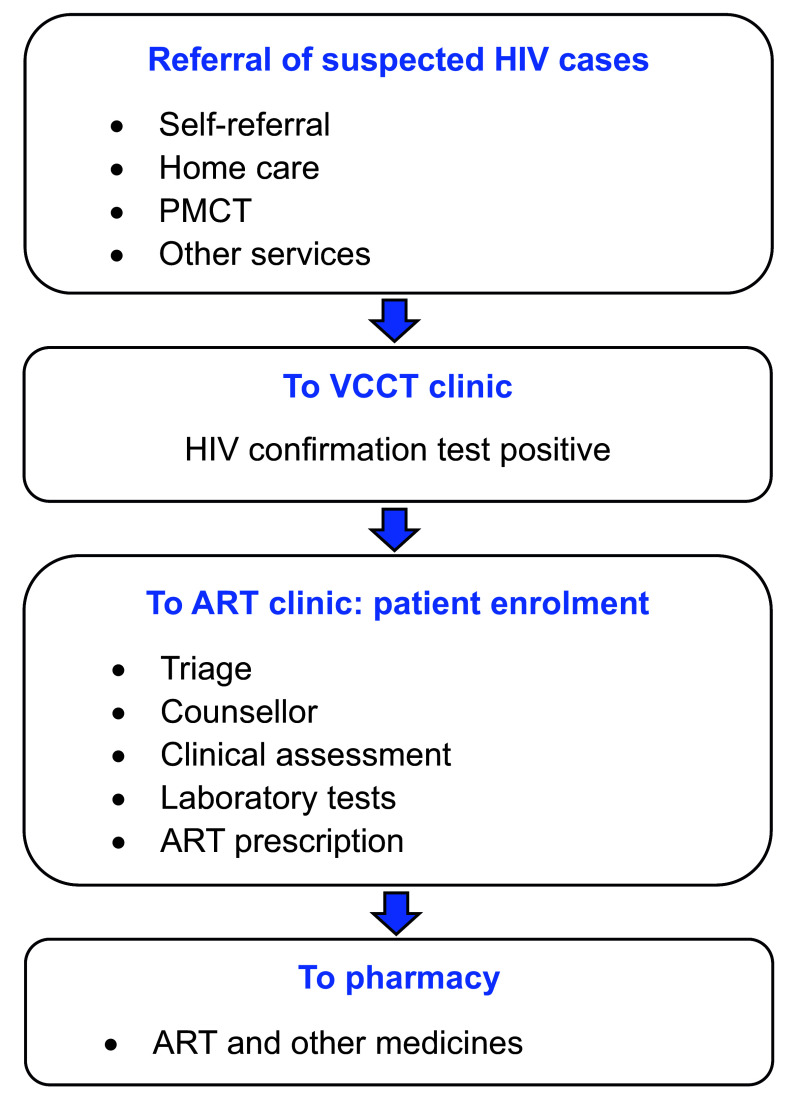
Pathways to diagnosis and enrolment in ART in Cambodia

**Fig. 1** shows the typical care pathway of HIV patients in Cambodia. Individuals suspected to have HIV are referred to voluntary confidential counselling and testing (VCCT) sites for confirmation of their status. Individuals may be referred from home care or community-based health-care services from programmes such as those aiming at prevention of mother-to-child transmission (PMCT); individuals can also self-refer. Once an HIV diagnosis is confirmed, newly diagnosed cases are referred to an ART clinic for treatment. Kampong Thom has three ART clinics, which are part of a national network of 69 clinics and health facilities that enrol people newly diagnosed with HIV in ART and that contribute data to the country’s ART surveillance system.

[**insert** Figure 1]

### Study design and data collection

This study is a secondary analysis of data collated by the National Center for HIV/AIDS Dermatology and STDs about people living with HIV who enrol in ART at clinics across Cambodia. Data from paper-based forms are entered into the database monthly by data officers at provincial and municipal health departments. Patient-level information captured by the system includes the confirmatory HIV test result and status of ART enrolment, previous and current treatment history, referral route, clinical stage of HIV, other diagnoses and treatment for opportunistic infections, as well as sociodemographic characteristics.

Data from 2014 to 2023 were extracted from the ART database for the three clinics in Kampong Thom province: Baray Santuk, Kampong Thom and Stoung. All newly diagnosed cases who engaged with one of these three ART clinics for treatment during this period were included in the analysis.

For the purposes of this analysis, late ART initiation was represented as a categorical variable (yes or no). Cases were categorized as late ART if they did not receive ART on the same day as they were diagnosed with HIV. Categorical variables were also created to represent patient sociodemographic and clinical characteristics that were considered to be potential risk factors for late ART initiation. The patient’s age at HIV diagnosis was grouped into one of four categories: 15–24 years, 25–34 years, 35–49 years and ≥ 50 years. Disease status was categorized using WHO stages I (infection), II (asymptomatic), III (symptomatic) and IV (AIDS/progression of HIV to AIDS). (*8*) Other variables for risk factors included sex (male, female), marital status (single, married, divorced/widowed, unknown), employment status (employed, unemployed, unknown), level of educational attainment (none, primary, secondary/higher, unknown), source of referral for VCCT (self-referral, home care/community, PMCT or other) and the ART clinic attended. In addition, cases were categorized according to their risk behaviours for HIV.

### Statistical analysis

Socioeconomic and clinical characteristics were summarized in the form of frequency and percentages for categorical variables and mean and standard deviation (SD) for continuous variables. We used univariate logistic regression models to estimate crude odds ratios (ORs) for the association between risk factors and late ART initiation. Multivariate logistic regression with adjusted ORs was used to identify which factors were independently associated with late ART initiation. Univariate and multivariate analyses were restricted to those diagnosed from 2019 onwards to reflect the impact of the introduction of the same-day policy on initiation of ART. A *P*-value of < 0.05 was considered to be statistically significant.

Statistical analyses were conducted using STATA version 17 (StataCorp, College Station, TX, USA).

## Results

**Fig. 2:**
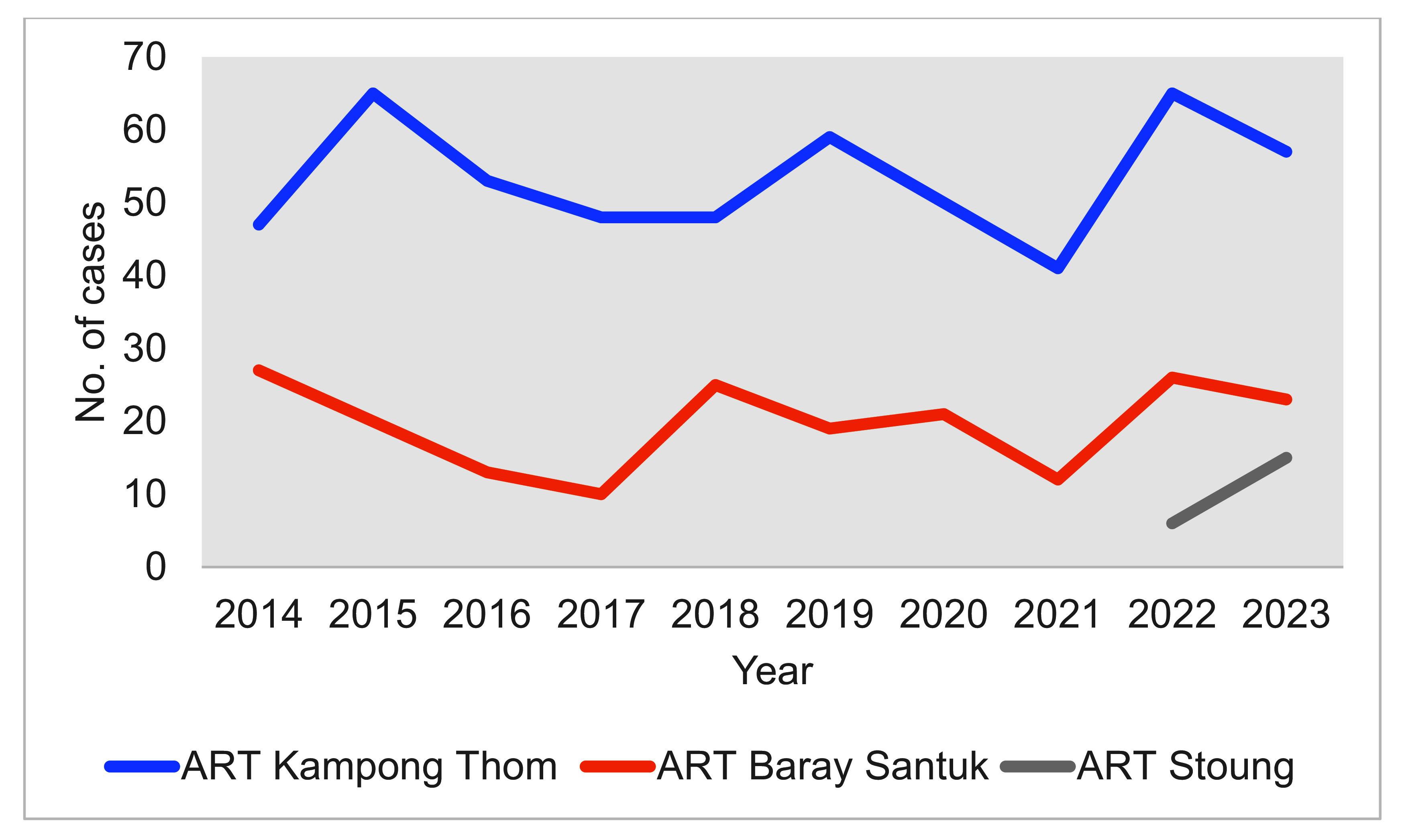
Number of newly diagnosed cases of HIV enrolled in the three ART clinics in Kampong Thom, Cambodia, 2014–2023 (*N* = 741)

A total of 741 patients newly diagnosed with HIV attended the three ART clinics in Kampong Thom province between 2014 and 2023. Two thirds of cases (511, 68.9%) were seen at the provincial hospital (ART Kampong Thom) (**Fig. 2**).

[**insert** Figure 2]

Of the 741 patients, 423 (57.1%) were female. The mean age at diagnosis was 45.7 years (SD: 10.2), with half falling within the 35–49-year age group (*n* = 408, 55.1%). Among the 591 whose marital status was known, 443 (75.0%) were married; 504 of 704 (71.6%) were employed. Of the 687 cases with data about educational attainment, around half (336, 48.9%) had primary education, and one third (215, 31.3%) had no formal education. Nearly three quarters (514/723, 71.1%) were in WHO stage IV (AIDS/progression of HIV to AIDS) at diagnosis. The majority of patients (602, 81.2%) were not in a high-risk category, and two thirds (470, 63.4%) had referred themselves for HIV diagnosis or confirmation; another third (236, 31.9%) were referred for confirmation by PMCT services (**Table 1**).

**Table 1:** Sociodemographic and clinical characteristics of patients newly diagnosed with HIV enrolled in three ART clinics in Kampong Thom province, Cambodia, 2014–2023 (*n* = 741)

Characteristic	Newly diagnosed cases of HIV	
	No.	Percentage
**Sex**		
**Male**	**318**	**42.9**
**Female**	**423**	**57.1**
**Mean (SD) age at diagnosis**	**45.7 (10.2)**	
**Age group (years)**		
**15–24**	**15**	**2.0**
**25–34**	**71**	**9.6**
**35–49**	**408**	**55.1**
** ≥ 50**	**247**	**33.3**
**Marital status**		
**Single**	**61**	**8.2**
**Married**	**443**	**59.8**
**Divorced/widowed**	**87**	**11.8**
**Unknown**	**150**	**20.2**
**Occupation**		
**Unemployed**	**200**	**27.0**
**Employed**	**504**	**68.0**
**Unknown**	**37**	**5.0**
**Education level**		
**No formal education**	**215**	**29.0**
**Primary**	**336**	**45.3**
**Secondary or higher**	**136**	**18.4**
**Unknown**	**54**	**7.3**
**Residence**		
**Kampong Thom province**	**678**	**91.6**
**Other province**	**62**	**8.4**
**ART site**		
**Kampong Thom**	**511**	**68.9**
**Baray Santuk**	**196**	**26.5**
**Stoung (commenced in 2022)**	**34**	**4.6**
**WHO stage**		
**I (infection)**	**16**	**2.2**
**II (asymptomatic)**	**80**	**10.8**
**III (symptomatic)**	**113**	**15.2**
**IV (AIDS/progression to AIDS)**	**514**	**69.4**
**Unknown**	**18**	**2.4**
**Type of patient (by risk behaviour)**		
**General population**	**602**	**81.2**
**Female entertainment worker**	**36**	**4.9**
**Men who have sex with men**	**78**	**10.5**
**Other^a^**	**25**	**3.4**
**VCCT referral source**		
**Self-referral**	**470**	**63.4**
**Home care and community**	**24**	**3.2**
**PMCT**	**236**	**31.9**
**Other^b^**	**11**	**1.5**
**Late ART initiation**		
**Yes**	**415**	**56.0**
**No**	**326**	**44.0**
**Year of diagnosis**		
**2014–2018**	**356**	**48.0**
**2019–2023**	**385**	**52.0**
**Median (range) days to ART initiation**		
**2014–2018**	**28 (0–2016)**	
**2019–2023**	**0 (0–96)**	

ART: antiretroviral therapy; PMCT: prevention of mother-to-child transmission; SD: standard deviation; VCCT: voluntary confidential counselling and testing.

^a^ This category includes patients notified for testing because they were partners of someone who was HIV-positive, pregnant, used or injected drugs, or transgender.

^b^ This category includes referral from a tuberculosis programme or other route.

Data were not available for all variables; denominators therefore vary across categories such as education level, employment status and WHO stage.

**Fig. 3:**
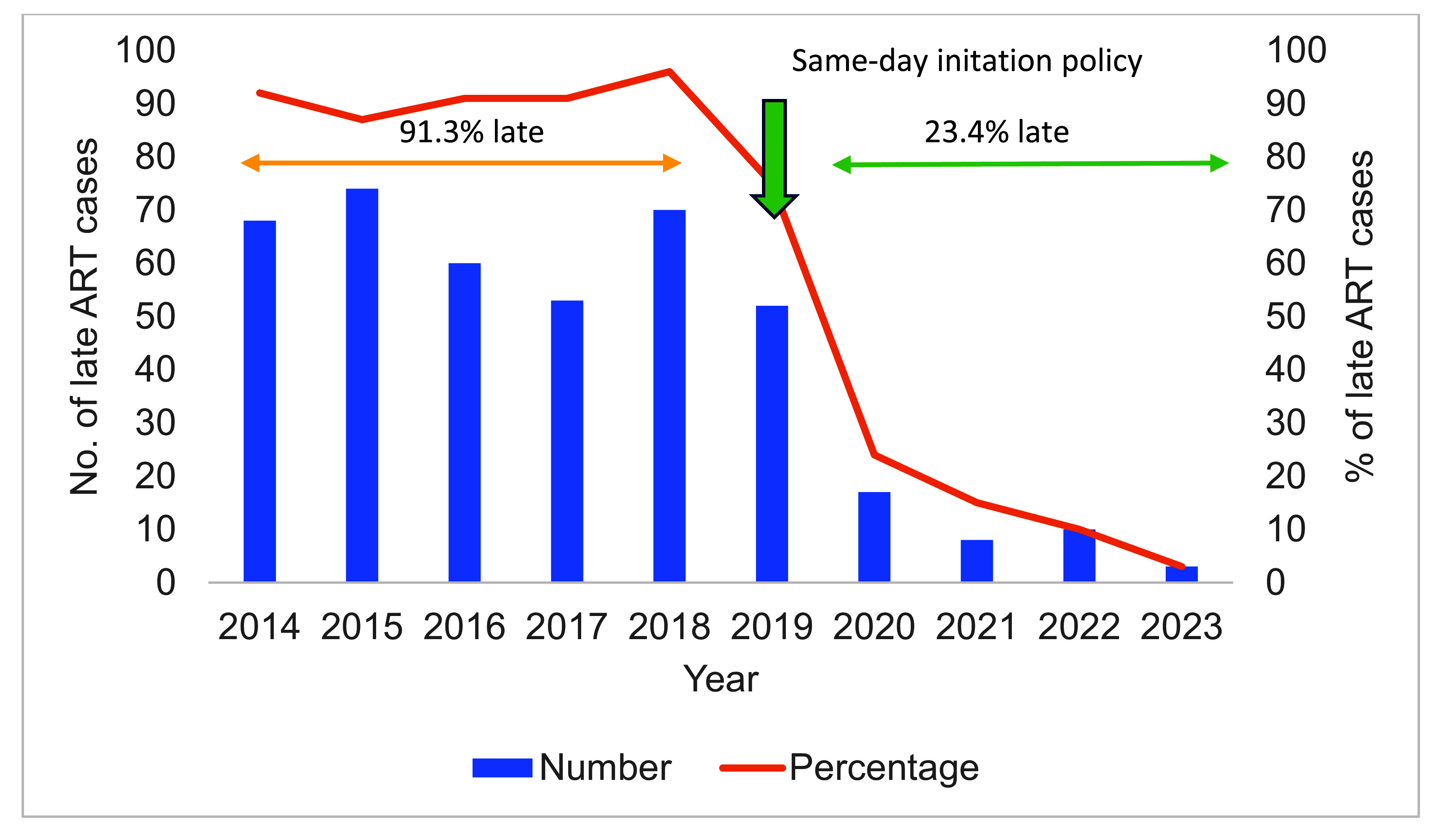
Number and percentage of people newly diagnosed with HIV starting ART late (>1 day after diagnosis) in Kampong Thom province, 2014–2023 (*N* = 741)

During the study period, the number of patients newly diagnosed with HIV who enrolled at ART clinics remained relatively stable (**Fig. 2**). Around half of enrolments (356, 48.0%) occurred during 2014–2018, and the other half during 2019–2023 (385, 52.0%). Overall, 415 (56.0%) patients experienced late ART initiation (> 1 day). Between 2014 and 2018, 325/356 (91.3%) people newly diagnosed with HIV did not start treatment on the same day compared with 90/385 (23.4%) between 2019 and 2023 (**Fig. 3**). The median time to ART initiation for those diagnosed during 2014–2018 was 28 days (range: 0–2016 days); it was 0 days (range: 0–96 days) for those diagnosed during 2019–2023 (**Table 1**). The number and proportion of people with late ART initiation decreased rapidly after 2019, reaching 3% in 2023 (**Fig. 3**).

[**insert** Figure 3]

During the 5 years from 2019 to 2023, females were overrepresented in the group who had delayed ART (65.5% [59/90] vs 34.4% [31/90], *P* = 0.095). Late initiation was more common among those aged 35–49 years than among those aged > 50 years (64.4% [58/90] vs 32.2% [29/90], *P* = 0.019), and among those who were employed vs unemployed (75.3% [67/89] vs 24.7% [22/89], *P* = 0.048) (**Table 2**). In multivariate analyses, factors independently associated with an increased likelihood of late ART initiation included receiving care at the Baray Santuk ART clinic (adjusted OR: 3.94, 95% confidence interval [CI]: 1.99–7.81, *P* < 0.001) and year of diagnosis. Late ART initiation was significantly lower in 2023 compared with 2019 (adjusted OR: 0.01, 95% CI: 0.00–0.02, *P* < 0.001).

**Table 2:** Univariate and multivariate analysis of factors associated with late ART initiation (> 1 day after diagnosis) after implementation of the same-day ART policy among patients newly diagnosed with HIV,  Kampong Thom province, Cambodia, 2019–2023 (*n* = 385)

Characteristic	Late ART^a^	Crude OR (95% CI)	*P*	Adjusted OR (95% CI)	*P*
**Sex**					
**Male**	**31 (34.4)**	**1**	**Reference**	**1**	**Reference**
**Female**	**59 (65.5)**	**1.52 (0.93–2.48)**	**0.095**	**1.73 (0.85–3.53)**	**0.128**
**Age at diagnosis (years)**					
**15–24**	**0 (0.0)**	**–**	**–**	**–**	**–**
**25–34**	**3 (3.3)**	**0.59 (0.16–2.12)**	**0.427**	**1.90 (0.26–13.6)**	**0.520**
**35–49**	**58 (64.4)**	**2.10 (1.27–3.49)**	**0.004**	**2.05 (0.98–4.28)**	**0.056**
** ≥ 50**	**29 (32.2)**	**1**	**Reference**	**1**	**Reference**
**Marital status**					
**Single**	**6 (9.0)**	**1**	**Reference**	**1**	**Reference**
**Married**	**51 (77.2)**	**1.38 (0.54–3.52)**	**0.496**	**1.90 (0.27–3.63)**	**0.990**
**Divorced/widowed**	**9 (13.6)**	**1.07 (0.34–3.37)**	**0.899**	**0.72 (0.16–3.23)**	**0.670**
**Occupation**					
**Unemployed**	**22 (24.7)**	**1**	**Reference**	**1**	**Reference**
**Employed**	**67 (75.3)**	**1.72 (1.0–2.95)**	**0.048**	**1.61 (0.75–3.46)**	**0.221**
**Education level**					
**No formal education**	**24 (28.2)**	**1**	**Reference**	**1**	**Reference**
**Primary education**	**47 (55.2)**	**1.41 (0.80–2.47)**	**0.226**	**1.82 (0.80–4.16)**	**0.151**
**Secondary or higher**	**14 (16.4)**	**0.85 (0.41–1.78)**	**0.678**	**1.30 (0.46–3.63)**	**0.614**
**Residence**					
**Kampong Thom**	**85 (94.4)**	**0.47 (0.15–1.49)**	**0.203**	**0.23 (0.03–1.61)**	**0.141**
**Other**	**5 (5.6)**	**1**	**Reference**	**1**	**Reference**
**ART site**					
**Kampong Thom**	**49 (54.4)**	**1**	**Reference**	**1**	**Reference**
**Baray Santuk^b^**	**36 (40.0)**	**2.33 (1.40–3.90)**	**0.001**	**3.94 (1.99–7.81)**	** < 0.001**
**Stoung**	**5 (5.6)**	**0.91 (0.33–2.53)**	**0.870**	**1.23 (0.36–4.20)**	**0.737**
**WHO stage**					
**I (infection)**	**0 (0.0)**	**–**	**–**	**–**	**–**
**II (asymptomatic)**	**7 (7.8)**	**0.59 (0.24–1.41)**	**0.242**	**0.65 (0.12–3.25)**	**0.620**
**III (symptomatic)**	**14 (15.5)**	**0.63 (0.33–1.21)**	**0.174**	**0.58 (0.16–2.05)**	**0.400**
**IV (AIDS/progression to AIDS)**	**69 (76.7)**	**1**	**Reference**	**1**	**Reference**
**Type of patient (by risk behaviour)**					
**General population**	**75 (83.3)**	**1**	**Reference**	**1**	**Reference**
**Female entertainment worker**	**3 (3.3)**	**0.21 (0.06–0.72)**	**0.013**	**0.30 (0.06–1.45)**	**0.136**
**Men who have sex with men**	**9 (10.0)**	**0.29 (0.14–0.63)**	**0.002**	**0.48 (0.18–1.31)**	**0.156**
**Other**	**3 (3.4)**	**0.31 (0.09–1.07)**	**0.065**	**1.18 (0.02–1.62)**	**0.127**
**VCCT referral type**					
**Self-referral**	**81 (90.0)**	**0.91 (0.41–1.99)**	**0.820**	**1.48 (0.54–4.01)**	**0.438**
**Other route**	**9 (10.0)**	**1**	**Reference**	**1**	**Reference**
**Year confirmed**					
**2019**	**52 (57.8)**	**1**	**Reference**	**1**	**Reference**
**2020^b^**	**17 (18.9)**	**0.10 (0.04–0.22)**	** < 0.001**	**0.80 (0.03–0.18)**	** < 0.001**
**2021^b^**	**8 (8.9)**	**0.05 (0.02–0.14)**	** < 0.001**	**0.04 (0.01–0.13)**	** < 0.001**
**2022^b^**	**10 (11.1)**	**0.03 (0.01–0.08)**	** < 0.001**	**0.02 (0.01–0.07)**	** < 0.001**
**2023^b^**	**3 (3.3)**	**0.01 (0.00–0.03)**	** < 0.001**	**0.01 (0.00–0.02)**	** < 0.001**

CI: confidence interval; OR: odds ratio; VCCT: voluntary confidential counselling and testing.

^a^ Values are *n*(%).

^b^ Statistically significant factor.

## Discussion

Before 2019, less than 10% of people newly diagnosed with HIV in Kampong Thom province started ART on the day their diagnosis was confirmed. This proportion rose dramatically following the introduction of the same-day ART policy in 2019, and by 2023, only 3.2% of people newly diagnosed with HIV had a delayed start to ART. The median time to ART was also significantly reduced by this policy. However, uniformly high rates of same-day initiation were not observed at all three ART sites in the province.

This study highlights the positive impact of implementation of the same-day ART policy in reducing the time to ART in people with HIV, a policy aligned with the objectives of WHO’s HIV treatment guidelines, which recommend initiating treatment as soon as possible. Other countries have also experienced improvements in the time-to-ART initiation after changes in policy. (*9*) In Jamaica, for example, same-day ART initiation increased from 37% to 51% between 2015 and 2019, following the introduction of the Treat All strategy. (*10*) Our study adds to the evidence that such strategies can be implemented and can successfully increase the proportion of patients newly diagnosed with HIV who start treatment immediately.

The clinical benefits of initiating ART immediately upon diagnosis have been demonstrated by several studies. One study in Rwanda showed that, on average, patients who began ART on the same day as diagnosis achieved better viral suppression than those who started ART 1–7 days or more than 7 days post-enrolment. (*11*) Other studies have suggested that same-day ART initiation may be associated with a higher loss to follow up, a potentially negative outcome and a finding that underscores the need for enhanced patient support and follow up. (*12*, *13*) Thus, the evidence regarding the impact of same-day ART initiation on retention in care is inconsistent: several observational studies have reported that same-day ART initiation may be associated with an increased loss to follow up, but others, particularly randomized controlled trials, have found the opposite – that is, same-day ART can improve engagement and retention in care. This discrepancy has been attributed to differences in study designs and selection biases. (*14*) The ensuing debate has underscored the need for studies that avoid such pitfalls by beginning follow up at the point of HIV testing and when patients are linked to care, rather than from the point of ART initiation. Although our study was not designed to directly assess the impact of same-day ART on retention outcomes, we adopted this approach and enrolled participants at the time of diagnosis.

Cambodia’s success may partly be due to VCCT and ART clinics being located at the same hospital and to VCCT staff actively referring patients to the ART clinics, which make access easier for patients and reduce the likelihood that individuals leave before attending the ART clinic. In this study, late ART initiation was more prevalent among those attending the Baray Santuk ART clinic than those attending the clinic in Kampong Thom. The subsequent review of these findings with HIV programme managers revealed that the Baray Santuk clinic does not have staff who are dedicated to HIV care, whereas the Kampong Thom clinic has multiple staff who are dedicated to delivering this care. Other studies have demonstrated that the success of same-day ART initiation is contingent upon the presence of skilled and committed personnel capable of addressing patients’ immediate needs following diagnosis. Insufficient staffing may hinder clinics’ ability to successfully implement same-day ART initiation, resulting in delayed ART. (*15*)

This study has several limitations. First, the surveillance data used in this analysis – which depend on those who collect the data and on those who enter these data at the ART sites – may vary in completeness, consistency and reliability across sites and over time. Second, the lack of complete patient data about clinical parameters, such as CD4 count, viral load, and signs and symptoms, limited our ability to fully characterize patients’ health at the time of their HIV diagnosis and thus assess a wider range of factors associated with late ART initiation. Moreover, because longitudinal follow-up data about treatment continuity were not collected, only trends in the timing of ART initiation could be described and conclusions could not be drawn about the comparative retention benefits of early versus delayed ART initiation. Finally, there could have been biases in the recording of the characteristics of the high-risk groups. For example, men who have sex with men and female entertainment workers may not disclose, for example, their sexual orientation or occupation, which means individuals will be misclassified in the analysis.

In conclusion, this study has demonstrated the feasibility of implementing a same-day ART initiation policy, even in a low-resource setting. The introduction of this policy in 2019 led to a significant improvement in the time to treatment for people newly diagnosed with HIV in Kampong Thom province. However, successful implementation depends on having staff dedicated to providing HIV care to ensure that ART can be offered on the same day as diagnosis. Further research is needed to assess the clinical impact of starting treatment on the same day in Kampong Thom, specifically to determine whether patients remain in HIV treatment and care, and whether same-day treatment initiation leads to improved clinical outcomes.

## References

[1] Global HIV & AIDS statistics – fact sheet [website]. Geneva: Joint United Nations Programme on HIV and AIDS; 2024. Available from: https://www.unaids.org/en/resources/fact-sheet, accessed 25 September 2024.

[2] HIV and AIDS: key facts [website]. Geneva: World Health Organization; 2025. Available from: https://www.who.int/news-room/fact-sheets/detail/hiv-aids, accessed 2 April 2024.

[3] Guidelines for managing advanced HIV disease and rapid initiation of antiretroviral therapy, July 2017. Geneva: World Health Organization; 2017. Available from: https://iris.who.int/handle/10665/255884, accessed 4 March 2025. 29341560

[4] Guidelines for the use of antiretroviral agents in adults and adolescents with HIV. Rockville: Office of AIDS Research, National Institutes of Health; 2025. Available from: https://clinicalinfo.hiv.gov/en/guidelines/hiv-clinical-guidelines-adult-and-adolescent-arv/initiation-antiretroviral-therapy, accessed 19 November 2025.

[5] Hung CC, Phanuphak N, Wong CS, Olszyna DP, Kim TH. Same-day and rapid initiation of antiretroviral therapy in people living with HIV in Asia. How far have we come? HIV Med. 2022 Oct;23(S4) Suppl 4:3–14. 10.1111/hiv.13410 36254390

[6] Despite impressive treatment results, Cambodia’s HIV response must address inequalities affecting children and young key populations [website]. Phnom Penh: National Center for HIV/AIDS Dermatology and STDs; 2023. Available from: https://www.nchads.gov.kh/documents_post/despite-impressive-treatment-results-cambodias-hiv-response-must-address-inequalities-affecting-children-and-young-key-populations/, accessed 26 September 2024.

[7] Standard operating procedures: same-day HIV PReP delivery by community based organizations for key populations in Cambodia. Phnom Penh: National Center for HIV/AIDS Dermatology and STDs; 2022. Available from: https://www.nchads.gov.kh/wp-content/uploads/2022/01/Finalizedwithsigned-CBO-PrEP-SOP_en.pdf, accessed 26 September 2024.

[8] Annex 10: WHO clinical staging of HIV disease in adults, adolescents and children. In: Consolidated guidelines on the use of antiretroviral drugs for treating and preventing HIV infection: recommendations for a public health approach, 2nd ed. Geneva: World Health Organization; 2016. Available from: https://iris.who.int/handle/10665/208825, accessed 21 October 2025.

[9] Onoya D, Sineke T, Hendrickson C, Mokhele I, Maskew M, Long LC, et al. Impact of the test and treat policy on delays in antiretroviral therapy initiation among adult HIV positive patients from six clinics in Johannesburg, South Africa: results from a prospective cohort study. BMJ Open. 2020 Mar 25;10(3):e030228. 10.1136/bmjopen-2019-030228 32213514 PMC7170559

[10] Cushnie A, Reintjes R, Figueroa JP, Artama M. Trends and factors associated with initiation of HIV treatment among PLHIV in Jamaica, 2015-2019. PLoS One. 2023 May 26;18(5):e0265468. 10.1371/journal.pone.0265468 37235603 PMC10218740

[11] Murenzi G, Kim HY, Shi Q, Muhoza B, Munyaneza A, Kubwimana G, et al. Association between time to antiretroviral therapy and loss to care among newly diagnosed Rwandan people living with human immunodeficiency virus. AIDS Res Hum Retroviruses. 2023 May;39(5):253–61. 10.1089/aid.2022.0023 36800896 PMC10171964

[12] Bakari HM, Alo O, Mbwana MS, Salim SM, Ludeman E, Lascko T, et al. Same-day ART initiation, loss to follow-up and viral load suppression among people living with HIV in low- and middle-income countries: systematic review and meta-analysis. Pan Afr Med J. 2023 Nov 27;46:92. 10.11604/pamj.2023.46.92.40848 38405092 PMC10891376

[13] Joseph Davey D, Kehoe K, Serrao C, Prins M, Mkhize N, Hlophe K, et al. Same-day antiretroviral therapy is associated with increased loss to follow-up in South African public health facilities: a prospective cohort study of patients diagnosed with HIV. J Int AIDS Soc. 2020 Jun;23(6):e25529. 10.1002/jia2.25529 32510186 PMC7277782

[14] Labhardt ND, Brown JA, Sass N, Ford N, Rosen S. Treatment outcomes after offering same-day initiation of human immunodeficiency virus treatment–how to interpret discrepancies between different studies. Clin Infect Dis. 2023 Oct 13;77(8):1176–84. 10.1093/cid/ciad317 37229594 PMC10573746

[15] Kerschberger B, Boulle A, Kuwengwa R, Ciglenecki I, Schomaker M. The impact of same-day antiretroviral therapy initiation under the World Health Organization treat-all policy. Am J Epidemiol. 2021 Aug 1;190(8):1519–32. 10.1093/aje/kwab032 33576383 PMC8327202

